# Addressing malnutrition among children in routine care: how is the Integrated Management of Childhood Illnesses strategy implemented at health centre level in Burundi?

**DOI:** 10.1186/s40795-019-0282-y

**Published:** 2019-03-05

**Authors:** Manassé Nimpagaritse, Catherine Korachais, Georges Nsengiyumva, Jean Macq, Bruno Meessen

**Affiliations:** 1 Institut National de Santé Publique, Avenue de l’Hôpital n°3/BP, 6807 Bujumbura, Burundi; 2 Health Economics Unit, Department of Public Health, Institute of Tropical Medicine, Nationalestraat 155, 2000 Antwerp, Belgium; 3 Université Catholique de Louvain, Institut de Recherche Santé et Société, Clos Chapelle-aux-Champs, 30 boîte 3016 -1200, Bruxelles, Belgium

**Keywords:** Malnutrition, IMCI, Health Centre, Burundi, Implementation research

## Abstract

**Background:**

The Integrated Management of Childhood Illness (IMCI) strategy was adopted in Burundi in 2003. Our aim was to evaluate to what extent the malnutrition component of the IMCI guidelines is implemented at health facilities level.

**Methods:**

We carried out direct observations of curative outpatient consultations for children aged 6–59 months in 90 health centres selected randomly. We considered both the child and the health worker (HW) as units of analysis and used bivariate analysis to explore characteristics of HWs associated with tasks systematically or never performed.

**Results:**

A total of 514 consultations carried out by 145 HWs were observed. Among the 250 children under two years, less than 30% were asked questions on breastfeeding. None of them had all seven nutrition-related questions asked to their caregivers and none of the 200 children over the age of two years had all five nutrition-related questions asked to their caregivers. Only 13 cases (3%) had all of the six examinations/tasks (weight, height/length, mid-upper arm circumference, oedema, filling in and discussing the growth curve and calculating the weight for height z-score) performed as part of their care. 393 cases (76%) reported that they had not being given any nutrition advice.

With regards to HWs, among 99 of them who had received children under two, only 21 (21.2%)[14.2–30.5%) systematically asked the question regarding ‘ongoing breastfeeding’.

Only 56 (38.6%)[31–46.9%] weighed or discussed the weight taken prior the consultation for each child they reviewed, only 38 (26.2%)[19.6–34.1%] measured the height/length or discussed it for each child reviewed and 23 (15.9%)[10.7–22.8%] performed (systematically?) the WHZ-score.

More than 50% never gave nutrition advices to any child reviewed.

HWs who daily manage severe acute malnutrition were the most likely to systematically ask the question regarding ‘ongoing breastfeeding’ and to perform a ‘weight examination’. Those who had not received supervision visit on the topic of malnutrition predominantly never performed a ‘weight examination’. The ‘height/length’ examination’ was predominantly performed by female HWs and those who have ‘contract with the government.

**Conclusion:**

This study has found poor compliance by HWs to IMCI in Burundi. This indicates that a substantial proportion of children do not receive early and appropriate care, especially that pertaining to malnutrition. This alarming situation calls for strong action by actors committed to child health in the country.

**Trial registration:**

Clinical Trials.gov Identifier: NCT02721160; March 2016 (retrospectively registered).

## Background

Under-five mortality remains a huge problem for sub-Saharan African countries, with an average under-five mortality rate of 79 deaths per 1000 live births in 2016 [1]. Preventable infectious diseases such as pneumonia, diarrhea and malaria remain leading causes of death among children under five years and account for one third of all under-five deaths [1]. Many of these deaths occur in children whose immune systems are already weakened by malnutrition—globally, nearly half of all deaths among children under five are attributable to malnutrition [1–3].

Individual health interventions shown to be effective in reducing child mortality include exclusive breastfeeding, improved vaccination coverage, oral rehydration therapy, treatment of pneumonia and early treatment of malaria in endemic areas [4–7]. However, most children at first-level health facilities have multiple and overlapping morbidities, which can make diagnosis and treatment difficult for the health workers (HWs). It gradually became clear to health care planners in the mid-1990s, that an approach integrating management of the most important health problems in childhood was needed to achieve better health outcomes. The World Health Organization (WHO), in collaboration with the United Nations Children’s Emergency Fund (UNICEF) and other agencies, developed a strategy known as the Integrated Management of Childhood Illnesses (IMCI).

The IMCI strategy aims at substantially reducing deaths due to diarrhea, pneumonia, malaria, measles, and malnutrition among children under five years old [8, 9]. It includes three main components [6, 1) improvement in the case management skills of HWs through provision of locally adapted guidelines on IMCI and activities to promote their use; (2) improvement in the overall health care system required for effective management of childhood illnesses and (3) improvement in family and community health care practices. The main strength of the strategy is that it guides HWs through a systematic evaluation of the conditions which lead to the greatest morbidity and mortality in children, with economically affordable diagnostic tools and medicines and with the active involvement of parents in the care and follow-up of their children.

The IMCI guidelines define steps for assessing, classifying, treating, and referring sick children; counseling the child’s caregiver and vaccinating children. A central tenet of IMCI is to organize the diagnosis/classifications of sick children on signs and symptoms; this allows implementation even by staff with limited medical skills.

Since its introduction, studies have demonstrated that IMCI could effectively improve quality of care for children and reduce child mortality when the three components were introduced and implemented concurrently and adequately [10]. A recent Cochrane systematic review on IMCI [11] concluded that the use of the IMCI strategy may lead to fewer deaths among children under five, but may have little or no effect on nutritional status when the community component is inadequately implemented.

Whether or not the malnutrition component of IMCI guidelines is implemented by HWs in the curative consultations of children under five is the focus of this study.

To our knowledge, there have been few studies describing in detail HWs’ assessments of children with regards to their nutritional status in the context of a weakened health care system and high prevalence of chronic malnutrition. This article presents HW performance during routine clinical practice in rural health centres in Burundi .

The structure of this article is as follows. First, a contextualization of the study with background information on IMCI and the health system in Burundi. Next, an overview of the analytical tool and data. The findings of the study are presented and discussed in the next two sections. The article concludes with recommendations for policymakers and researchers.

## Context

Burundi is one of the poorest countries in the World, with a GDP per capita estimated in 2016 at PPP $ 777 [12]. The civil war during the 1990s significantly affected the health system, especially with regards to human resources. Medical doctors are a rare resource in Burundi (in 2010, the ratio was 1 doctor per 19,231 inhabitants) [13] – under such constraints, IMCI is a particularly relevant strategy.

Burundi adopted the two first components of the IMCI strategy in 2003. At that time, Burundi had an infant mortality rate of 129 per thousand live births, one of the highest in sub-Saharan Africa. Severe acute malnutrition among children under five years varied by region and ranged between 1.1 and 4.1% [14]. The third component (improvement in family and community health care practices) was adopted in 2006.

Under IMCI, it was expected that all children who presented to health facilities would be routinely assessed for nutritional status and treated when identified as malnourished.

So far, practices of HWs in routine care with regards to malnutrition in Burundi has not been studied in a systematic way. It should be a priority as Burundi is facing continuing high rates of infant mortality and high caseloads of acute malnutrition. In 2017, the infant mortality rate was still at 47 per thousand live births and the prevalence of severe and moderate acute malnutrition was respectively at 1 and 4% [15].

## Methods

This study builds on an impact evaluation of the Performance Based Financing (PBF) scheme applied to malnutrition in Burundi’s health centres. A full presentation of the research has been published elsewhere [16]. The research protocol was approved by ethical committees in Burundi and at the University of Antwerp, as well as the Institutional Review Board of the Institute of Tropical Medicine, Belgium. For this paper, we use data coming from the health facility baseline survey, which was conducted in September 2014 in 90 health centres selected randomly among 193 health centres that offer services to treat both moderate acute malnutrition (MAM) and severe acute malnutrition (SAM).

### Survey tool and data

Within each of the 90 randomly selected health centres, trained surveyors performed direct observation of six curative consultations for children aged 6–59 months coming for a first-time visit in a given illness episode. The aim was to evaluate the HWs current practices with particular attention to the assessment of the nutritional status of the child, but also assessing their history taking and examination skills and subsequent actions taken, including counseling. We examined them along fourteen routine tasks adapted from the IMCI guidelines [17, 18] (see Table 1).

**Table 1:** IMCI-based tasks to be performed

*Danger signs*		
1	Check for ability to drink or breastfeed	
2	Check whether the child vomits everything	
3	Check whether the child has had one or more seizures	
*Main Symptoms*		
4	Cough **or** Difficulty breathing	
5	Check for diarrhea	
6	Check for fever	
7	Check for ear problems	
*Others*		
8	Check for malnutrition	
		*Nutrition questions* Ongoing breastfeeding (BF) (<2y only)
		Episodes of BF since the morning/previous evening (<2y only)
		Appetite
		Other complimentary foods normally eaten
		Age complimentary foods were started
		What has s/he eaten since yesterday (24 h recall)
		Recent change in diet
		*Nutrition examinations/tasks*
		Weight
		Height/length
		MUAC
		Oedema
		Growth curve
		WHZ
9	Check for Anemia	
10	Check for vaccination status	

*Source: adapted from WHO 2014*

Based on IMCI and national malnutrition guidelines [19], a list of questions was included to assess the history taking. They covered issues which should be discussed in any general paediatric consultation in primary health care settings in low-income countries, such as breastfeeding, other complimentary foods normally eaten, …. A similar process was used for the physical examination. Parameters relevant to nutrition such as weight, height/length, mid-upper arm circumference (MUAC), presence of oedema, calculation of the weight-for-height z-score (WHZ) and evaluation of the growth curve were considered as areas to be assessed.

The eight surveyors had a four-day training regarding the objectives of observations and how to fill the questionnaire in correctly. The training included interactive lectures, small group exercises, video tutorials and role plays. They had both a written and practical exam on the fifth day of the training and each surveyor pre-tested the questionnaire in a pilot health centre. The group of surveyors was made up predominantly of nurses.

To minimise the bias in the behaviour of the HW in the presence of the surveyor, surveyors had to memorise a script to explain the objectives of the survey tool to the HW. They emphasised that this was not a test and the impartial nature of their observations. HWs were also informed that the survey was independent from the qualitative assessments quarterly done under the PBF scheme: the evaluation would neither affect their employment as a nurse in the HC nor their income. Surveyors were told to observe the HWs’ interactions with children and their caregivers without interfering. Both HWs and caregivers were explained the objectives of the study and had to give their written consent for participation before the start of any observation.

For the history taking section of the questionnaire, the surveyors were instructed to record whether a question from the set list was asked and then to write the caregiver’s response. With regards to the anthropometric measures, the surveyors were asked to record either if the HWs themselves had taken a specific measure and/or had referred to it or discussed it with the caregiver during the course of the consultation.^1^

In addition, surveyors had to collect information from the HWs themselves regarding their contracts, education, in-service training, supervision, etc.

### Analysis

We carried out descriptive analyses on our sample of paediatric consultations. This was assessed as sufficient to understand to what extent the malnutrition component of the IMCI guidelines is implemented in health facilities in Burundi. Data were double-entered, cleaned and validated using data entry screens in CSPro. Analyses were conducted using Stata (version 14.1).

We first used the child as a unit of analysis. We calculated the proportion of children for whom all questions and examinations related to nutrition were asked and performed and those for whom no questions or examinations were asked or performed. Then, we used health workers as a unit of analysis to evaluate HWs’ current practices in the implementation of the malnutrition component of the IMCI guidelines. To assess their performance, we calculated the proportion of HWs who asked questions and performed examinations related to nutrition ‘systematically’ (for every child) and ‘never’ (for any child) during observed consultations. The same path was followed in terms of nutritional advice given in curative consultations.

Finally, bivariate analysis was used to explore associations between HWs performance and their characteristics (such as gender, age, contract type, etc.) with regards to the malnutrition component of IMCI guidelines. We used the Pearson’s chi-squared test to compare HWs for some of their characteristics and adjusted the *p*-value for multiple comparisons using the Bonferroni correction.

## Results

A total of 514 consultations were observed.^2^ These consultations were carried out by 145 health workers. As stated above, our results focus mainly on the observed history and examination.

### Nutrition history and examinations of children

Among the 514 children observed during a consultation, 456 (89%) were asked about their age. Of the 450 children for whom we have responses, there were 250 children under the age of two and 200 children over the age of two years. Seven questions were relevant for children under two years (including questions on breastfeeding) and five relevant for children above two years (not including questions on breastfeeding).

For the 250 children under two, only 76 caregivers (30%)[24.5–35.9%] had been asked the question on ‘ongoing breastfeeding’ and 15 caregivers (6%)[3–8.9%] were asked the question on ‘episodes of breastfeeding since the morning/previous evening’.

For none of the 250 children under the age of two, were all the seven nutrition questions asked to their caregivers and for 106 (43%)[36–48.2%] cases, none of these questions were asked. Similarly, for none of the 200 children over the age of two years, were all the five questions asked to their caregivers and for 93 (47%)[39.5–53.5%] of them, no question at all was asked.

There were six aspects of the physical examination relating specifically to the nutritional status of the child: weight, height/length, MUAC, oedema, filling in and/or discussing the growth curve and calculating the WHZ. In total, 13 (3%)[0,9%-6.9] cases had all of the six examinations and tasks performed as part of their care and 211 (41%)[33.2–49.3%] cases had none of the examinations and tasks performed. Table 2 gives more details on questions asked and examinations performed.

**Table 2:** Nutrition questions asked and examinations performed

	Cases asked(%)	95% CI
Nutrition questions		
Cases <2y asked all seven questions*(n = 250)*	0 (0)	
Cases <2y asked none of the seven questions*(n = 250)*	106 (43)	[36,0-48,2]
Cases >2y asked all five questions*(n = 200)*	0 (0)	
Cases >2y asked none of the five questions*(n = 200)*	93 (47)	[39,5-53,5]
Ongoing breastfeeding (BF) (<2y only: n = 250)	76 (30)	[5, 35]
Episodes of BF since the morning/previous evening (<2y only: n = 250)	15 (6)	[3,0-8,9]
	*(n = 514)*	
Appetite	226 (44)	[37,9-50,3]
Other complimentary foods normally eaten	74 (15)	[4, 19]
Age complimentary foods were started	16 (3)	[1, 5]
What has s/he eaten since yesterday (24 h recall)	17 (4)	[2,0-5,4]
Recent change in diet	6 (1)	[0,5-2,6]
Nutrition examinations/tasks	*(n = 513)*	
Cases undergoing all six examinations/tasks	13 (3)	[0,9-6,9]
Cases undergoing none of the examinations/tasks	211 (41)	[33,2-49,3]
Weight	268 (52)	[43,5-60,7]
Height/length	165 (32)	[24,0-41,4]
MUAC	153 (30)	[3, 38]
Oedema	64 (13)	[8,0-19,0]
Growth curve	37 (7)	[7,2–100]
WHZ	95 (19)	[4, 26]

### Characteristics of observed health workers

Table 3 presents the characteristics of the surveyed HWs: age, gender, employer, diploma, in-service trainings they had received, their duties and the supervision visits they had received (from the health district management team) with regards to the topic of malnutrition. All observed HWs were nurses with predominantly a minimum of two years nursing training (61%). Most HWs were employed by the government (85%) and more than half of them had not received any in-service training (55%) since the end of their studies. 70% of the HWs had the management of MAM as part of their activities and 61% had not received supervision the last six months.

**Table 3:** Characteristics of the health workers (*N* = 145)

Characteristics	Number (%)
Age	
*< 35 years*	99 (68)
*> 35 years*	46 (32)
Gender	
*male*	76 (52)
*female*	69 (48)
Employer	
*government*	123 (85)
*health centre*	22 (15)
Diploma	
*A3* ^1^	*88 (61)*
*A2* ^2^ *and above*	*57 (39)*
Years since last in-service training	
*<=1 year*	101 (70)
*> 1 year*	44 (30)
Have received training on growth monitoring and promotion	
*Yes*	21 (14)
*No*	124 (86)
Have received training on screening and management of acute malnutrition	
*Yes*	60 (41)
*No*	85 (59)
Self-declared need of further training in growth monitoring and promotion	
*Yes*	52 (36)
*No*	91 (64)
Self-declared need of further training in screening and management of acute malnutrition	
*Yes*	89 (62)
*No*	54 (38)
Management of moderate acute malnutrition is part of the activities of the health worker	
*Yes*	102 (70)
*No*	43 (30)
Management of severe acute malnutrition is part of the activities of the health worker	
*Yes*	73 (50)
*No*	72 (50)
Have received supervision visit on the topic of malnutrition in the last six months	
*Yes*	57 (39)
*No*	88 (61)

^1^two years of nursing training

^2^four years of nursing training

### Performance of health workers in relation to nutrition questions

As shown in Table 4, among the 99 HWs who had received children under two, 21 (21.2%)[14.2–30.5%) systematically^3^ asked the question regarding ongoing breastfeeding while 49 (49.5%) [39.6–59.4%] never asked this question during any observed consultation. Moreover, among the 145 HWs observed, only 26 (17.9%)[12.5–25.1%] systematically asked about any change in appetite and six (4.1%)[1.9–9.0%] about any other complimentary foods normally eaten. These questions were never asked by an important share of the HWs, respectively 37(25.5%)[19.%-33.3] and 98 (67.6%)[59.5–74.8%].

**Table 4:** Performance of health workers in relation to nutrition questions

**Nutrition questions**	**Systematically** ^**1**^ **asked**	**Never asked**
Ongoing breastfeeding (BF)	21 (14,5%) [9.6%–21,3%]	78 (53,8%) [45,6%-61,8%]
Episodes of BF since the morning/previous evening	5 (3,5%) [1.46%–8,1%]	131 (90,3%) [84,3%-94,2%]
Appetite	26 (17,9%) [12,5%-25,1%]	37 (25,5%) [19,0%-33,3%]
Other complimentary foods normally eaten	6 (4,1%) [1,9%-9,0%]	98 (67,6%) [59,5%-74,8%]
Age complimentary foods were started	0	129 (89,0%) [82,7%-93,2%]
What has s/he eaten since yesterday (24 h recall)	0	130 (89,7%) [83,5%-93,7%]
Recent change in diet	0	139 (95,9%) [91,0%-98,2%]

^1^for every child

Finally, none of the HWs systematically asked the remaining questions regarding the age complimentary foods were started, what the child had eaten since the day before (24-h recall) and whether there was a recent change in their diet.

### Performance of health workers in relation to nutrition examinations

Among the 145 observed HWs, 56 (38.6%)[31.0–46.9%] systematically weighed or discussed the weight taken prior the consultation, 38 (26.2%)[19.6–34.1%] systematically measured the height/length or discussed it, and 23 (15.9%)[10.7–22.8%] systematically performed the WHZ-score. Only one HW (0.7%) systematically discussed the growth curve for each child. Moreover, only eight HWs (5,5%) [2, 7–10] systematically sought oedema.

On the contrary, most of them did not perform any of those examinations for all children reviewed in curative consultation: 51(35.2%)[27.8–43.4%] for the weight, 90(62.1%)[53.8–69.7%] for height/length, 111 (76.6%)[68.9–82.8%] for the WHZ-score, 117 (80,7%) [73,4%-86,4%] for oedemaand 127 (87,6%) [81,1%-92,1%] for the growth curve. Table 5 gives more details.

**Table 5:** Performance of health workers in relation to nutrition examinations

**Nutrition examinations/tasks**	**Systematically** ^**1**^ **performed**	**Never performed**
Weight	56 (38,6%) [31,0%-46,9%]	51 (35,2%) [27,8%-43,4%]
Growth curve	1 (0,7%) [0,0–4%]	127 (87,6%) [81,1%-92,1%]
Height/length	38 (26,2%) [19,6%-34,1%]	90 (62,1%) [53,8%-69,7%]
MUAC	25 (17,2%) [11,9%-24,4%]	91 (62,8%) [54,5%-70,3%]
Oedema	8 (5,5%) [2, 7–10]	117 (80,7%) [73,4%-86,4%]
WHZ	23 (15,9%) [10,7%-22,8%]	111 (76,6%) [68,9%-82,8%]

### Performance of health workers in relation to nutrition advice

Ten (6.9%)[3.7–12.4%] HWs systematically gave nutrition advice and 76 (52.4%)[44.2%-61.%] never gave nutrition advice to any child reviewed.

Results displayed in Table 6 and Table 7 suggest that HWs with the ‘management of severe acute malnutrition’ as part of their daily activities were the most likely to ask systematically the question regarding ‘ongoing breastfeeding’ with a difference of 16.0 percentage points [0.0–32.2]. On the other hand, those who do not have this activity among their attributions seem to be the most likely to never ask this question in any observed consultation (with a difference of 23.2 percentage points [3.6–42.8]. Compared to males, female HWs predominantly systematically asked the question on appetite (12.8 percentage points [0.2–25.3]).

**Table 6:** Characteristics of health workers^1^ with tasks systematically performed

Characteristics		Ongoing BF(*n* = 99)	Appetite (*n* = 145)	Weight (n = 145)	Height/length (n = 145)	Whz (n = 145)
Age	< 35	22,7%	18,2%	36,3%	29,2%	15,1%
	> = 35	18,2%	17,4%	43,4%	19,5%	17,3%
	Difference	4,5%pts.(−12,9%-21,9%)	0,8%pts.(− 12,8–14,4%)	7,1%pts.(−24,3-10,1%)	9,7%pts.(−5,8-25,2%)	2,2%pts.(− 15,2-10,7%)
	*p*-value (of difference)	0.606	0,908	0,416	0,217	0,733
Gender	Male	20%	11,8%	31,5%	17,1%	10,5%
	Female	22,4%	24,6%	46,3%	36,2%	21,7%
	Difference	2,4%pts.(− 14,0-19,0%)	12,8%pts.(0,2-25,3%)	14,7%pts.(− 1,1-30,7%)	19,1%pts.(4,9-33,3%)	11,2%pts.(−0,7-23,1%)
	p-value (of difference)	0,768	0,045	0,068	0,008	0,065
Employer	Health Centre	21,4%	22,7%	27,2%	9,0%	4,5%
	State	21,2%	17,1%	40,6%	29,2%	17,8%
	Difference	0,2%pts.(−23,3-23,8%)	5,6%pts.(−11,9-23,3%)	− 13,3%pts. (−35,7-8,9%)	−20,1%pts.(−40,1-0,1%)	− 13,3pts(−30,0-3,3%)
	p-value (of difference)	0,983	0,527	0,238	0,047	0,116
Diploma	A3	13,0%	19,3%	38,6%	27,2%	17,0%
	A2 and more	28,3%	15,8%	38,5%	24,5%	14,0%
	Difference	−15,2%pts.(−31,4-0,9%)	3,5%pts.(−16,4-9,4%)	0,0%pts.(− 16,5-16,4%)	2,7%pts.(− 17,5-12,1%)	3,0%pts.(− 15,3-9,3%)
	p-value (of difference)	0,065	0,591	0,996	0,719	0,630
had not received any in-service training since the end of their studies	Yes	15,7%	20%	31,2%	21,2%	11,2%
	No	27,1%	15,3%	47,6%	32,3%	21,5%
	Difference	11,4%pts.(−4,9-27,7%)	4,6%pts.(−17,3-8,1%)	16,4%pts.(0,4-32,3%)	11,0%pts.(−3,4–25,5%)	10,2%pts.(−1,7-22,3%)
	p-value (of difference)	0,169	0,474	0,043	0,134	0,092
Management of MAM is part of the activities of the HW	Yes	23,6%	16,7%	42,1%	27,4%	15,6%
	No	14,8%	20,9%	30,2%	23,2%	16,2%
	Difference	8,8%pts.(−27,2-9,6%)	4,3%pts.(−9,6-18,1%)	−11,9%pts. (−29,4-5,5%)	4,1%pts.(−20,0-11,7%)	0,5%pts.(−12,6 − 13,8%)
	p-value (of difference)	0,345	0,544	0,180	0,602	0,929
Management of SAM is part of the activities of the HW	Yes	28,8%	15,1%	47,9%	32,8%	20,5%
	No	12,8%	20,8%	29,1%	19,4%	11,1%
	Difference	16,0%pts.(−32,2–0%)	5,8%pts.(−6,8–18,4%)	18,7%pts.(−34,5—2,9%)	13,4%pts.(−27,8-0,9%)	9,4%pts.(−21,4-2,5%)
	p-value (of difference)	0,051	0,369	0,020	0,066	0,121
Supervision	Yes	21,6%	17,5%	31,5%	22,8%	14,0%
	No	20,9%	18,2%	43,1%	28,4%	17,0%
	Difference	0,6%pts.(−17,6-16,3%)	0,6%pts.(−12,3-13,6%)	11,6%pts.(−4,7-27,9%)	5,6%pts.(−9,2-20,4%)	3,0%pts.(− 9,3-15,3%)
	p-value (of difference)	0,939	0,922	0,163	0,457	0,630

^1^*P*-values were adjusted for multiple comparison: after Bonferroni correction, p-values were no more significant

**Table 7:** Characteristics of health workers^1^ with tasks never performed

Characteristics		Ongoing BF (n = 99)	Appetite (n = 145)	Weight (n = 145)	Height/length (n = 145	Whz (n = 145)
Age	< 35	51,5%	21,2%	37,3%	59,5%	77,7%
	> = 35	45,4%	34,7%	30,4%	67,3%	73,9%
	Difference	6,0%pts.(−15,2-27,3%)	-13,5pts (−28,8-1,7%)	6,9%pts.(−9,9-23,8%)	7,7%pts.(−24,9-9,3%)	3,8%pts.(−11,1–18,8%)
	p-value (of difference)	0,574	0,082	0,418	0,371	0,612
Gender	Male	50%	28,9%	42,1%	69,7%	77,6%
	Female	48,9%	21,7%	27,5%	53,6%	75,3%
	Difference	1,0%pts.(−21,1–19,1%)	−7,2%pts. (−21,5-7,1%)	−14,5%pts. (−30,1-1,0%)	−16,1% pts. (−31,9—0,2%)	2,2%pts.(−16,2-11,7%)
	p-value (of difference)	0,920	0,323	0,067	0,046	0,749
Employer	Health centre	28,5%	22,7%	22,7%	77,2%	81,8%
	State	52,9%	26,0%	37,3%	59,3%	75,6%
	Difference	−24,3%pts. (−52,8-4,1%)	3,2%pts.(−23,3-16,7%)	−14,6%pts. (−36,5 − 7,1%)	17,9%pts.(−4,2-40,0%)	6,2%pts.(−13,2-25,7%)
	p-value (of difference)	0,092	0,746	0,186	0,112	0,530
Diploma	A3	45,2%	20,4%	37,5%	60,2%	77,2
	A2 and more	54,3%	33,3%	31,5%	64,9%	75,4%
	Difference	9,0%pts.(−11,0-29,1%)	12,8%pts.(−1,7-27,4%)	5,9%pts.(−22,0-10,2%)	4,6%pts.(− 11,7-21,0%)	1,8%pts.(−16,1–12,5%)
	p-value (of difference)	0,373	0,083	0,469	0,573	0,800
had not received any in-service training since the end of their studies	Yes	50,9%	18,7%	38,7%	70%	85%
	No	47,9%	33,8%	30,7%	52,3%	66,1%
	Difference	3,0%pts.(−23,2 − 17,0%)	15,0%pts.(0,8-29,3%)	-7,9%pts.(− 23,7-7,8%)	-17,6% pts.(−33,5—1,8%)	−18,8%pts. (−32,5—5,1%)
	p-value (of difference)	0,763	0,038	0,320	0,029	0,007
Management of MAM is part of the activities of the HW	Yes	45,8%	24,5%	33,3%	56,8%	73,5%
	No	59,2%	27,9%	39,5%	74,4%	83,7%
	Difference	13,4%pts.(−9,0–35,8%)	3,3%pts.(−12,3-19,1%)	6,2%pts.(−11,0-23,4%)	17,5%pts.(0,2-34,8%)	10,1%pts.(−5,0 − 25,4%)
	p-value (of difference)	0,238	0,670	0,478	0,047	0,188
Management of SAM is part of the activities of the HW	Yes	38,4%	27,3%	32,8%	50,6%	68,4
	No	61,7%	23,6%	37,5%	73,6%	84,7
	Difference	23,2%pts.(3,6-42,8%)	3,7%pts.(−18,1–10,6%)	4,6%pts.(−11,1-20,3%)	22,9%pts.(7,3-38,5%)	16,2%pts.(2,4–29,9%)
	p-value (of difference)	0,020	0,604	0,563	0,004	0,021
Supervision	Yes	45,9%	22,8%	50,8%	63,1%	73,6%
	No	51,6%	27,2%	25%	61,3%	78,4%
	Difference	5,6%pts.(−15,1-26,4%)	4,4%pts.(−10,2-19,2%)	-25,8%pts. (−41,4—10,2%)	1,7%pts.(−18,2-14,6%)	4,7%pts.(−9,5-19,0%)
	p-value (of difference)	0,589	0,550	0,001	0,829	0,515

^1^P-values were adjusted for multiple comparison and after Bonferroni correction, p-values were no more significant

With regards to nutrition examinations, findings suggest that HWs who had received an in-service training since the end of their studies and those with the management of severe acute malnutrition as part of their daily activities were the most likely to systematically perform a weight examination (with a difference of respectively 16.4 percentage points [0.4–32.3] and 18.7 percentage points [2.9–34.5]). In addition, HWs who had not received supervision visit on the topic of malnutrition over the last six months predominantly never performed a weight examination (with a difference of 25.8 percentage points [10.2–41.4]).

The female HWs and those who have a contract with the government predominantly systematically performed a height/length examination (with a difference of respectively 19.1 percentage points [4.9–33.3] and 20.1 percentage points [0.1–40.1]).

On the other hand, male HWs, those who do not have the management of MAM or SAM as part of their activities and those who had not received any in-service training were most likely to never perform the height/length examination. Finally, HWs who do not have the management of SAM as part of their activities and those who had not received any in-service training since the end of their studies predominantly never calculated the WHZ-score.

Overall, none of the HWs systematically performed all the tasks related to malnutrition (questions and examinations).

## Discussion

The IMCI guidelines were developed with the knowledge that many primary health centres in low-income countries lack doctors. It is hoped that thanks to IMCI, nurses, clinical officers, community health workers, etc., who assess and manage sick children perform a sufficiently good level of care to generate real health benefits for the children. Our results cast doubt on such an outcome in Burundi: overall, our study found a consistently poor level of adherence on IMCI guidelines by HWs, which is likely to affect the quality of care for their patients. This low compliance is consistent with evaluations of IMCI case management practices in several other settings [20–23] and other studies in the field of quality of care for sick children [24]. This finding indicates a missed opportunity to benefit Burundi’s children given that some studies have reported that IMCI is associated with improvements in the quality of health care [25–27].

The IMCI guidelines include a malnutrition component. The importance of this being that it is within these curative consultations that screening can be carried out for malnutrition to aid early detection and treatment and indeed prevention if possible.

A nutritional history is essential in all paediatric consultations but is notoriously neglected by HWs in many primary health care settings in low-income countries [28, 29]. IMCI guidelines require that all children under two years get a feeding assessment; this especially as breastfeeding has already been shown to reduce mortality in infants and young children [4, 30–32]. Our study found that only a small proportion of children were asked about their diet and only few HWs consistently asked about (maximum) four out of the seven questions relating to malnutrition. Anthropometric measurements are also essential. However most of the children in our study missed the opportunity of accurate nutritional assessment and detection of malnutrition. Weight was systematically recorded by about 38% of HWs, but anthropometric measurements such as weight alone or height/length alone are themselves not useful unless they are interpreted according to resp. height or age.

Our exploratory analyses also established a link between lack of weight measurement and supervision. This confirms again how IMCI implementation rests on supervision [33]. This strongly suggests the need to train the district teams on IMCI, focusing on improving IMCI guidelines knowledge and supervision skills to implement plans and monitor progress [34].

There were worryingly few children who had oedema assessed (13%), a factor which can completely change the diagnosis of a child from a normal z-score or MUAC or MAM to SAM, which is a diagnosis requiring prompt and comprehensive treatment, especially in light of the fact that SAM children with oedema have higher risks of mortality [35, 36]. The poorest performance was observed with regards to the number of HWs who discussed the growth curve.

Child health interventions including breastfeeding, nutrition counseling about complementary feeding and growth monitoring have been shown to prevent malnutrition and substantially reduce child mortality [4, 37]. Although exclusive and continued breastfeeding rates are high in Burundi, nutrition advice was frequently not given and growth monitoring not performed.

Overall, the results suggest that female health workers perform better than males on every task. We believe that this can be glued to the fact that in our culture, it is the women who care most about the children needs at home (breastfeeding, feeding them, getting them to vaccination, or in curative consultation in case of illness). It is quite possible that these mothers who are used to care children needs better investigate the problems of a child who is brought to curative consultation.

We also found that HWs who have a (permanent) contract with the government perform better, suggesting that the stability these contracts confer does not entail their motivation, on the contrary.

Overall, required tasks are not systematically performed (if ever), and most of the time consultations are limited to the presenting complaint; this could lead to incorrect diagnoses of the children’s condition and to potentially miss a serious and treatable disease. Such a failure in a context of high prevalence of malnutrition raises a lot of questions. One can imagine that the non-systematization of questions and physical examinations is linked to the fact that the tasks of the IMCI guidelines are numerous. Even these minimal tasks are not properly completed.

Our bivariate analysis suggests that being examined by the HW in charge of the activity of management of malnutrition increases the chance for the child to be weighed, while lack of training on monitoring growth and screening and management of malnutrition plays negatively on the weight-for-height/length z-score calculation.

These results suggest that a poorly implemented split of tasks is not conducive to malnutrition screening. Some rotation of staff within the different departments of the HC would probably increase staff versatility and hopefully, lead to a higher attention to malnutrition in routine consultation. While the low performance of HWs is frequently attributed to a lack of knowledge and skills, it appears that the diploma of HWs do not play a role in malnutrition screening. This raises questions about the initial training program of HWs with regards to malnutrition, but also about the strategies undertaken to improve HW performance [38]. Our findings also raise questions on the commitment of the HW to implement the malnutrition IMCI guidelines component. A possible cause could be that IMCI consultations take longer compared to routine consultations. Yet other results (not reported here) [39], obtained from administering vignettes to the same HWs at the end of the day’s activities (ideal conditions without pressure from the patient’s queue), suggest that the main problem is at the level of IMCI knowledge held by staff.

We believe that there is a need for complementary qualitative studies to investigate the possible factors associated to this low performance. One question of interest should be how HWs perceive the IMCI guidelines including the various tasks.

The recent Cochrane systematic review evaluated the effects IMCI strategy implementation in terms of death, nutritional status and quality of care [11]. However, the certainty of the evidence has been assessed as too low to allow an affirmative conclusion on whether or not IMCI has an effect on the way of HWs treat common illnesses. Our results are consistent with those of two studies focused on febrile children in Ghana [40] and Zambia [22]. In these two middle-income countries, the studies by Baiden et al. and Lunze et al. respectively also highlighted that adherence to IMCI case management guidelines were rather poor and below expectations. Our study complements their works by showing that the problem also arises for malnutrition, which is very rarely the presenting complaint in curative consultation.

Our study shows that the systematic integration of malnutrition screening into the ambulatory care package is problematic. In Burundi, management of acute malnutrition has for a long time been considered as the ‘turf’ of international NGOs. It could be interesting to explore whether other countries with a weak health system and a high prevalence of malnutrition present a similar pattern of limited integration.

Our study has limitations. First, the mere fact of being research subjects and the attention from the surveyors may have temporarily altered the behavior of the observed HWs. This could have changed their usual practices in curative consultation by trying to prove that they are doing their job properly.

If this effect has played any role, our results actually underestimate the depth of the problem.

Second, we were also unable to determine the adherence to IMCI guidelines between trained and untrained HWs on IMCI guidelines given that the period since the last training varies from one HC to another and according to staff changes. That said, some studies have reported a positive impact of IMCI training on quality of care [21, 41], however others found no effect [42, 43].

## Conclusion

This study has found poor compliance by HWs to IMCI guidelines in health centers of Burundi. This leads to a substantial proportion of children not receiving early and appropriate care, especially malnutrition care. The incomplete implementation of IMCI prevents Burundi from seizing the maximum benefits from the strategy in terms of child survival.

Our findings call for vigorous action, by the Ministry of Health of Burundi and all its partners committed to child health and involved in the IMCI strategy. Obviously, what has been done until now is not satisfactory. We believe that the country needs a multi-faceted approach to ‘save’ the IMCI strategy. Training has to be improved, but more consistent and more meaningful supervision is the key element needed to address this non-compliance.

Our study also rings an alarm for the international and national actors involved in the ‘free health care + performance-based financing‘strategy in Burundi [44]. Through the IMCI metric, our study captures a central component of quality of care at health centre level. The observed low compliance to IMCI shows that a serious general problem with quality of care persists, despite attention put upon quality through PBF quality checklists. Possible actions include a revision of how quality of care is measured under the national PBF program (see for example, [45]) and the embedment of PBF into a more comprehensive approach to the determinants of quality of care.

But the implications of our study go also beyond Burundi. As shown in our discussion, our findings are consistent with observations in other countries. There seems to be a serious problem with the implementation of the IMCI strategy in many low-income countries. This is very worrying as the strategy, designed for countries with high child mortality and low availability of medical staff, is the cornerstone of pediatric care for hundreds of millions of children.

## Abbreviations

GDP: Gross Domestic Product
HW(s): Health Worker(s)
IMC: Integrated Management of Childhood Illness
MAM: Moderate Acute Malnutrition
MUAC: mid-upper arm circumference
PBF: performance-based financing
PPP: Purchasing Power Parity
SAM: Severe Acute malnutrition
UNICEF: United Nations International Children’s Emergency Fund
WHO: World Health Organization
WHZ: Weight-for-Height Z-score

## Footnotes

1 Anthropometrics measures are most of the time performed prior to consultation by other HWs

2 We did not reach 540 observations because there were not enough cases in some health centres (10)

3 i.e. to every child received in consultation
